# Longitudinal Evaluation of Humoral Immunity and Bacterial and Clinical Parameters Reveals That Antigen-Specific Antibodies Suppress Inflammatory Responses in Active Tuberculosis Patients

**DOI:** 10.1155/2018/4928757

**Published:** 2018-07-04

**Authors:** Mamiko Niki, Takashi Yoshiyama, Yuji Miyamoto, Masao Okumura, Makoto Niki, Ken-ichi Oinuma, Yukihiro Kaneko, Sohkichi Matsumoto, Yuka Sasaki, Hideo Ogata, Hajime Goto, Shoji Kudoh, Yoshihiko Hoshino

**Affiliations:** ^1^Department of Bacteriology, Osaka City University Graduate School of Medicine, Abeno, Osaka 545-8585, Japan; ^2^Division of Respiratory Medicine, Fukujuji Hospital, Japan Anti-Tuberculosis Association, Matsuyama, Kiyose, Tokyo 204-8522, Japan; ^3^Department of Mycobacteriology, Leprosy Research Center, National Institute of Infectious Diseases, Aoba, Higashi-Murayama, Tokyo 189-0002, Japan; ^4^Department of Bacteriology, Niigata University Graduate School of Medicine, Niigata 951-8510, Japan

## Abstract

A novel tuberculosis vaccine to replace BCG has long been desired. However, recent vaccine trials focused on cell-mediated immunity have failed to produce promising results. It is worth noting that most commercially available successful vaccines rely on humoral immunity. To establish a basic understanding of humoral immunity against tuberculosis, we analyzed and evaluated longitudinal levels and avidity of immunoglobulin to various tuberculosis antigens compared with bacterial and clinical parameters during treatment. We found that levels of IgG antibodies against HrpA and HBHA prior to treatment exhibited a positive correlation with bacterial burden. Analysis of changes in CRP during treatment revealed an association with high levels of specific IgG and IgA antibodies against mycobacterial antigens. Levels of CRP prior to treatment were negatively associated with IgG avidity to CFP-10 and MDP1 and IgA avidity to HrpA, while IgA avidity to MDP1 and Acr exhibited a negative correlation with CRP levels after 60 days of treatment. These results may provide insight for the development of a novel tuberculosis (TB) vaccine candidate to induce protective humoral immunity against tuberculosis.

## 1. Introduction

Tuberculosis remains one of the most prevalent infectious diseases worldwide and is caused by *Mycobacterium tuberculosis* (Mtb). There were 1.4 million tuberculosis (TB) deaths and 10.4 million new TB cases in 2015. Approximately, one-third of the world's population is latently infected with Mtb, which represents a huge reservoir of future disease progression and transmission. Mtb is transmitted through the air from a person with active TB to a healthy individual. In addition, Mtb has no environmental or animal reservoirs and is believed to have coevolved with humans [1]. Therefore, an effective Mtb vaccine to prevent infection is the best strategy to eradicate tuberculosis.

The BCG vaccine has been the only available way to combat tuberculosis long before the development of antibiotics [2]. Although the BCG vaccine is effective for the prevention of children's disseminated tuberculosis, it has limited protective capacity on the development of adult pulmonary tuberculosis caused by the reactivation of persistent Mtb. With increasing numbers of cases involving HIV- and TB-coinfected people and multidrug-resistant TB, the development of a more effective vaccination strategy is sorely needed.

As Mtb is an intracellular pathogen, the activation of cell-mediated immunity (CMI) characterized by IFN-*γ*-producing CD4^+^ T cells is regarded as crucial for TB immunity [3]. Research on vaccines that induce CMI has dominated much of the conventional development effort, since antibody-mediated immunity has been considered to play little role in the outcome of Mtb infection [4]. To date, several candidate vaccines targeting the induction of CMI have reached clinical trials. One of these new vaccines, namely, MVA85A, is an attenuated vaccinia virus expressing Mtb antigen Ag85A, which induces high levels of antigen-specific CD4^+^ and CD8^+^ T cells in a murine model [5]. Although MVA85A achieved successful results in mouse models and phase I human clinical trials, the final outcome of the trial was found to exhibit no detectable improvement of protection against TB [6, 7]. Recently, a first-in-human phase I trial was conducted to investigate AERAS-422, a recombinant BCG overexpressing Mtb antigens and mutant perfringolysin derived from *Clostridium perfringens* [8]. However, high-dose AERAS-422 vaccination was found to elicit reactivation of the varicella-zoster virus (VZV), possibly due to negative regulation of immune control of latent VZV induced by the elevated IFN-*γ* production [9]. It is obvious that the conventional approach to targeting CMI against TB is insufficient.

In contrast to the research on CMI, many of the studies on humoral immune responses against Mtb antigens focused largely on their use in the diagnosis of TB, since some of the studies indicated that the serum antibody levels against Mtb antigens correlate with the degree of bacterial load [10, 11]. However, accumulating experimental evidence suggests that humoral immunity can modulate the immune response to intracellular pathogens [12–16]. In addition, studies on vaccines with protective efficacy based on antibody-mediated immunity against some of these pathogens have been reported [17–21]. Therefore, humoral immunity has been consistently highlighted as an important component of protective immune responses to Mtb [22]. As several reports have revealed a potential role of specific antibodies in host defense against Mtb [23–26], vaccination that induces Mtb-specific antibodies in the airway mucosa could be an effective strategy for protection against primary infection prior to Mtb entry into the lung.

In addition to the quantity of antibodies, the avidity of antibodies seems to be an important contributing factor to the protective capacity of vaccines. Antibody avidity is the functional affinity of multivalent antibody to bind multivalent antigens. It can be used to determine the net antigen binding force of a heterogeneous population of antibodies and has been used as a marker of B cell maturation during viral and bacterial infection [27–30]. In many infectious diseases including TB, elevated antibody avidity is observed in patients with chronic or severe conditions [31]. Although high-avidity antibodies are found to be crucial for the protection conferred by vaccines against many pathogens [32–35], there has been little investigation into the role of antibody avidity of anti-Mtb antibodies in protection against TB.

We previously demonstrated that serum levels of Mtb antigen-specific IgA, not IgG, correlated with clinical statuses of TB patients, suggesting that specific IgA antibodies could play a role in protection from disease [36]. In this study, we observed a relationship between clinical parameters related to TB severity and a change in antibody levels and antibody avidity to Mtb antigens, both before and after treatment.

## 2. Subjects and Methods

### 2.1. Participants

Patients of Fukujuji Hospital, Tokyo, Japan, were consecutively enrolled, after giving written informed consent, from April 2010 to March 2013. A total of 205 patients were recruited for this study. Patients were diagnosed as active-phase tuberculosis by clinical symptoms, chest X-ray images, and bacterial cultures. When blood samples both before and after treatment were available, they were included in the analysis. A total of thirty-three patients (age; 55.6 ± 16.4 yrs, male: 66.7%) were analyzed (Table 1). All patients took Japanese standard medications for tuberculosis (RFP + INH + EB(SM) + PZA for 2 months and RFP + INH for an additional 4 months) [37]. No patients dropped out of the treatment during the clinical course, and no patients were relapsed by the time of the analysis. There were no deceased patients during this analysis. The following information was obtained from all patients at the time of enrollment: history of prior TB disease, work history in any healthcare setting or recent exposure to a patient with active TB, and other TB risk factors, such as having immunodeficiency disorders or taking immunosuppressive drugs. We used the same inclusion/exclusion criteria as in a previous study [36]. Information on previous medical history and clinical signs and symptoms were also collected as previously described [36]. “Smear at entry” (entry = point of diagnosis before treatment) indicates the number of acid-fast bacilli inspected in the sputum smear taken at entry. The severity was subdivided as 0 (no acid-fast bacilli (AFB) on smear), ± (1-2 AFB per 300 fields), 1+ (1–9 AFB per 100 fields), 2+ (more than 10 AFB per 100 fields), and 3+ (more than 10 AFB per field). Several routine laboratory tests, including serum concentration of “C-reactive protein (CRP) at entry” and “CRP after 60 days” of treatment, were simultaneously performed. Blood sample collection was performed before treatment and after treatment for the analysis of immunoglobulin levels and avidity. The research protocol was approved by the Institutional Review Boards of Osaka City University Graduate School of Medicine, Osaka, Japan, and Fukujuji Hospital, Tokyo, Japan, and by the Research Ethics Committee of the National Institute of Infectious Disease, Tokyo, Japan.

### 2.2. Measurement of Serum Antibody Levels

Concentrations of IgG and IgA antibodies against Mtb were determined by ELISA using recombinant proteins as previously described with slight modification [36]. Ninety-six well microplates (Sumilon Type H, LMS, Tokyo, Japan) were coated with each recombinant antigen in bicarbonate buffer, pH 9.6 overnight at 4°C. The plates were blocked with phosphate buffered saline (PBS) containing 0.05% Tween 20 and 5% skim milk for 12 hr at 4°C and washed four times with PBS containing 0.05% Tween 20. Human serum samples diluted 1 : 200 in PBS containing 0.05% Tween 20 and 0.5% skim milk were then added in duplicate (IgG) or triplicate (IgA) to the antigen-coated wells and incubated for 12 hr at 4°C. After washing the wells, HRP-conjugated anti-human IgG or IgA antibodies were added at a 1 : 2000 or 1 : 1000 dilution, respectively. Following one-hour incubation at 37°C, the plates were washed four times before 100 *μ*l of SureBlue reserve TMB was added to each well. The reactions were stopped after 10 min by adding 50 *μ*l of 0.1 M HCl, and absorbance was measured at 450 nm using a Multiskan Spectrophotometer (Thermo Fisher Scientific, Yokohama, Japan). The results of the IgG-ELISA were expressed as absorbance at 450 nm, whereas results of the IgA-ELISA were expressed as ELISA-Index, *S*/(*B* + 3SD), where *S* is the average OD value of the triplicate test samples and *B* + 3SD [38] corresponds to the average OD value of the triplicate negative controls (*B*) plus three times the standard deviation (SD).

### 2.3. Avidity ELISA

Antibody avidity is a measure of the overall accumulated strength of the interaction between multiple antigenic epitopes and a multivalent antibody. For the measurement of the avidity of antibody, an incubation step with 7 M urea for 15 min after the serum incubation to elute the low-avidity antibodies was added to the ELISA assay procedure described above. Avidity indexes were obtained by calculating the ratio of the antibody levels measured by ELISA with and without urea treatment.

### 2.4. Reagents and Recombinant Protein Preparation

pET-21b, pET-22b, Luria-Bertani (LB) medium, and carbenicillin were from Sigma (St. Louis, MO, USA); isopropyl-1-thio-beta-D- galactopyranoside and Ni-NTA agarose were from Qiagen (Gaithersburg, MD, USA); skim milk was from Morinaga (Tokyo, Japan); horseradish peroxidase-conjugated anti-human IgG or IgA antibodies were from Dako (Carpinteria, CA, USA); SureBlue reserve TMB microwell peroxidase substrate was from KPL (Gaithersburg, MD, USA). Expression and purification procedures for recombinant mycobacterial antigens (ESAT-6, CFP-10, MDP1, Ag85A, Acr, HBHA, and HrpA [Acr2]) were described previously [36].

### 2.5. Statistical Analysis

Spearman's rank correlation coefficient was used to determine the correlation between ELISA values and the severity of clinical status values. All analyses were performed using online statistics calculators (http://www.socscistatistics.com/tests/Default.aspx, http://vassarstats.net/index.html, http://molpath.charite.de/cutoff/index.jsp). The threshold of significance was set at *p* < 0.05.

## 3. Results

### 3.1. Results of Clinical Parameters before and after Tuberculosis Treatment

We collected bacterial and clinical parameters as severity of smear at entry, CRP levels (mg/dl) at entry, and CRP levels (mg/dl) after 60 days of treatment (Table 1). We also measured levels of humoral immunity as IgG level, IgG avidity index, IgA level, and IgA avidity index (Supplemental Table 1).

### 3.2. Measurement and Comparison of Serum IgG Levels and IgG Avidity to Various Mtb Antigens before and after Treatment

**Figure 1:**
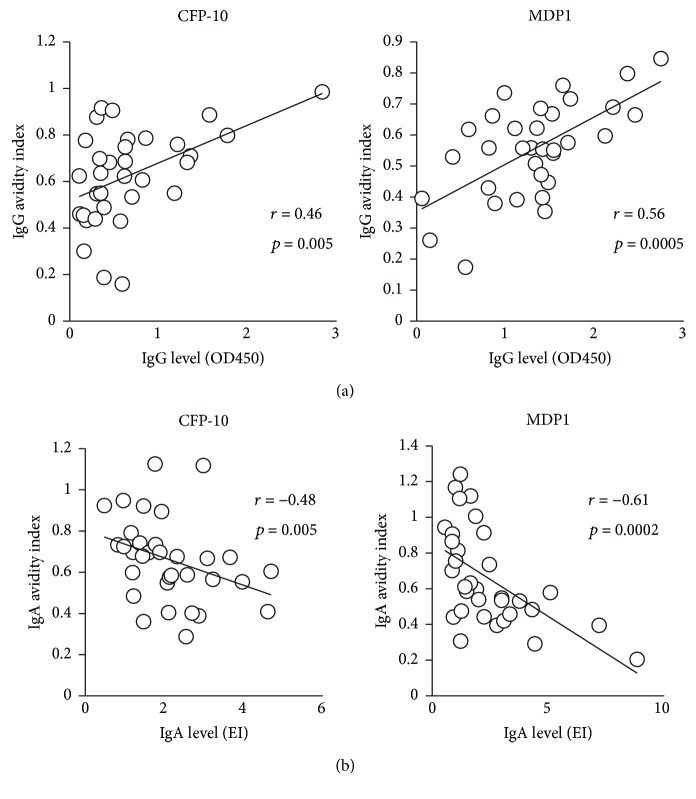
(a) Correlation between IgG levels and IgG avidity index against CFP-10 and MDP1 before treatment. (b) Correlation between IgA levels and IgA avidity index against CFP-10 and MDP1 before treatment.

**Figure 2:**
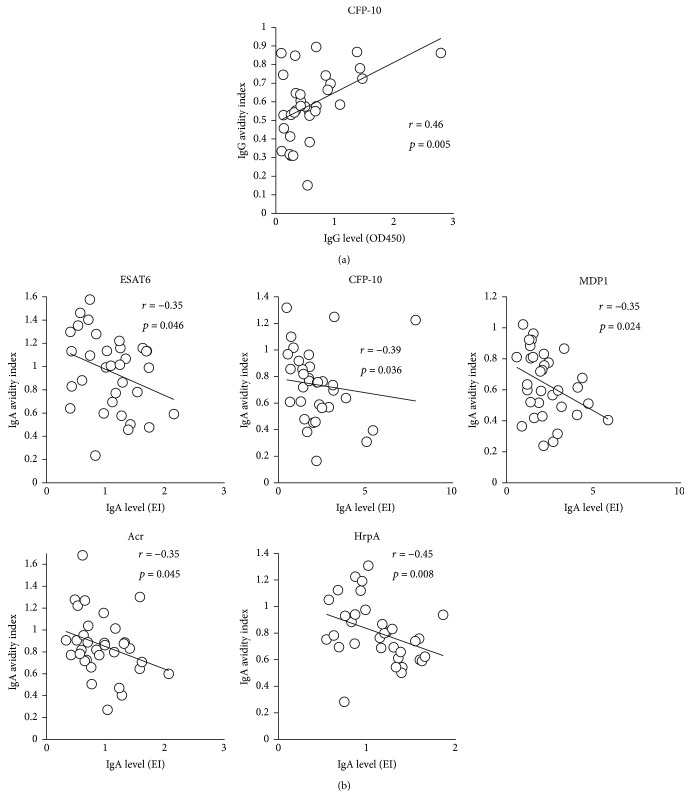
(a) Correlation between IgG levels and IgG avidity index against CFP-10 after treatment. (b) Correlation between IgA levels and IgA avidity index against 5 antigens after treatment.

We evaluated whether TB treatment affects serum antibody levels and antibody avidity indices for various Mtb antigens. A previous study showed that the levels of IgG against certain antigens decreased after the initiation of treatment [36], although it was also reported that the antibody response is heterogeneous and varies by individual, type of antigen, severity of the disease, and bacterial load [39, 40]. In our study, we observed a significant decrease in IgG levels against Acr and HrpA and in IgG avidity levels against MDP1 and Ag85A, during treatment (Supplemental Figure 1A–1B). On the other hand, neither IgA levels nor IgA avidity indices showed significant differences when compared before and after treatment (Supplemental Figure 2A–2B). Correlations between the change of serum antibody levels and antibody avidity levels during treatment were also analyzed. We found a statistically significant positive correlation between serum IgG levels and IgG avidity levels against CFP-10 and MDP1 before treatment (Figure 1(a)). Conversely, analysis of the relationship between serum IgA levels and IgA avidity against CFP-10 and MDP1 showed a negative correlation before treatment (Figure 1(b)). After treatment, only serum IgG level and avidity level against CFP-10 showed a positive correlation (Figure 2(a)), whereas almost all IgA tested was found to have a negative correlation between their antibody levels and the avidity levels (Figure 2(b)).

### 3.3. Analysis of the Relationship between Bacterial Load and Serum Mtb Antigen-Specific Antibody

**Figure 3:**
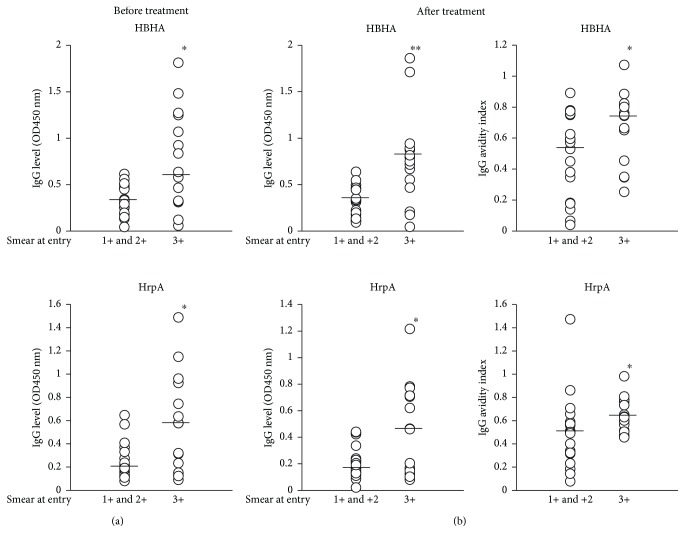
(a) Levels of serum IgG against HBHA and HrpA before treatment in smear at entry subgrouped between 1+ and 2+ and 3+. Vertical lines: mean values and ^∗^ *p* < 0.05. (b) Serum IgG levels and avidity indices against HBHA and HrpA after treatment in smear at entry subgrouped between 1+ and 2+ and 3+. Vertical lines: mean values and ^∗∗^ *p* < 0.01, ^∗^ *p* < 0.05.

To investigate whether the bacterial load affected the quantity or avidity of Mtb antigen-specific antibody, we compared the antibody levels and their avidity levels with “smear at entry” value. We found that HBHA- and HrpA-IgG levels before treatment showed a positive relationship with “smear at entry” (Figure 3(a)). We also observed that patients with high “smear at entry” scores gained high serum IgG levels and avidity levels against these antigens after treatment (Figure 3(b)). Meanwhile, neither the IgA levels nor IgA avidity levels were found to be associated with “smear at entry” values (data not shown).

### 3.4. Analysis of the Relationship between Serum CRP Level and Serum Mtb Antigen-Specific Antibody

**Figure 4:**
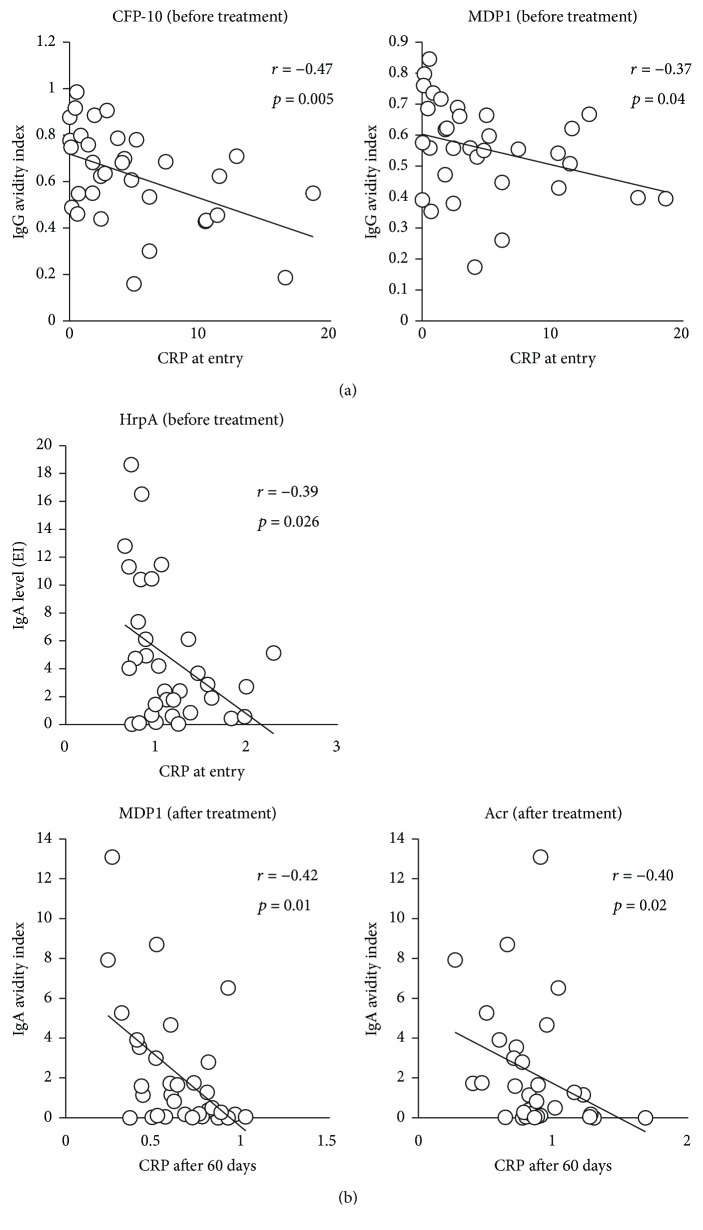
(a) Correlation between CRP at entry and IgG avidity index against CFP-10 and MDP1 before treatment. (b) Correlation between CRP at entry and IgA level against HrpA before treatment and correlation between CRP after 60 days and IgA avidity index against MDP1 and Acr after treatment.

To observe the association between antibody responses and the progression of disease, the antibody levels as well as avidity levels were compared to the serum CRP levels. We found that CFP-10 and MDP1 IgG avidity before treatment showed a reverse association with serum CRP levels at entry (Figure 4(a)). It was also found that HrpA-IgA level before treatment showed a negative correlation with CRP before treatment, and MDP1- and Acr-IgA avidity after treatment also showed a negative correlation with CRP after 60 days of treatment (Figure 4(b)).

## 4. Discussion

In this study, we evaluated the relationship between clinical parameters and antibody avidity to mycobacterial antigens. The aims of this study were (1) identification of the antigens that induce antibody production and maturation in TB patients and (2) evaluation of the correlation between serum antibody levels and antibody avidity to mycobacterial antigens and the host inflammatory response in TB progression. We tested 7 antigens that are highly immunogenic and are reported to be vaccine-candidate antigens. Acr is known to be a member of the dormancy regulon-encoded antigens. HrpA, also known as Acr2, is a member of the *α*-crystallin family in mycobacteria, which shares 30% homology with Acr, and is strongly upregulated following infection of macrophages [41]. Both antigens have been found to contribute to prolonged Mtb infection and disease progression. Ag85A is involved in mycobacterial cell wall assembly [42] and is primarily expressed during the early stages of infection, during which Mtb cells replicate rapidly and require synthesis of cell wall components. MDP1 is a mycobacterial histone-binding protein [43], which is reported to be strongly highly expressed during latent Mtb infection [44]. HBHA is a heparin-binding hemagglutinin protein and a mycobacterial surface-expressed adhesin, which is reported to be involved in extrapulmonary dissemination of Mtb [45] by enhancing adherence and phagocytosis of mononuclear phagocytes [46].

In this study, we found a significant decrease in IgG levels against Acr and HrpA and IgG avidity to MDP1 and Ag85A during treatment. In our previous study, we showed that MDP1 and Ag85A are expressed in Mtb cells inside tuberculous granuloma lesions in an asymptomatic subject and induced production of specific antibodies in latent TB patients [44]. In contrast to IgG responses, we did not find any significant differences in IgA antibody levels and IgA avidity to Mtb antigens when compared before and after treatment.

We found that IgG avidity to CFP-10 and MDP1 before treatment exhibited a negative association with serum CRP levels at entry. As IgG avidity is generally higher in chronically infected patients than patients with acute infection, IgG avidity to these antigens may reflect the duration of Mtb infection. Additionally, we observed that levels of IgG antibodies against HBHA and HrpA exhibited a positive relationship with “smear at entry,” and IgG avidity elevation during treatment was observed in patients with high IgG levels against these antigens before treatment. These findings indicate that patients with high bacterial burden produced higher levels of IgG against HBHA and HrpA. In agreement with our findings, several studies have indicated that serum IgG antibody levels against Mtb antigens correlate with the bacterial burden in active TB patients [10, 47, 48]. On the other hand, there were other studies showing that antibody levels do not always correspond to the bacterial load in TB patients [49, 50]. The inconsistency of these results may be due to the heterogeneity of antibody responses to Mtb antigens between individuals and among patients in different clinical stages [39, 40]. In experimental models, intravenous administration of IgG against mycobacterial lipoarabinomannan reduced bacterial load in the lung in mice intravenously infected with Mtb [25]. Another study using a mouse TB model revealed that intranasal administration of antigen 85B-HBHA fusion protein as a booster following BCG vaccination showed a significant reduction in bacterial load [51]. On the other hand, Mtb-reactive monoclonal antibodies increased uptake of Mtb cells by human lung epithelial cells [52]. These data suggest that the protective function of IgG may depend on the ability of the target antigen to induce sufficient IgG production. In addition, a recent study demonstrated that most humans with active TB exhibited lower serum IgG avidity to the Mtb cell surface [53]. Although these published data suggest a protective role for IgG in TB, further studies are needed for the identification of protective Mtb antigen epitopes and the induction of mature B cells that produce high-avidity antibodies crucial for the development of an effective vaccine.

As IgA in its secretory form is the main effector molecule of the mucosal immune system and serves as the first line of defense against pathogen invasion initiated at mucosal surfaces, we also investigated the role of IgA in TB progression [54]. We found that bacterial load did not affect serum IgA levels or IgA avidity to Mtb antigens. On the other hand, patients with elevated IgA levels against HrpA at the initial visit were found to exhibit lower CRP levels at entry. These results suggest that early induction of high levels of specific antibodies and avidity may suppress the inflammatory response. We have now confirmed that high levels of HrpA were significantly associated with lower serum CRP levels at entry in two different settings, namely, the current longitudinal study, as well as a previous cross-sectional study [36]. Moreover, we demonstrated that induction of antibody avidity to mycobacterial antigens may be associated with lower serum CRP levels, a marker of lower inflammatory status. Consistent with our results, several reports in both animal and human models showed that IgA provides early protection against Mtb infection. In mice, IgA-deficiency leads to increased susceptibility to intranasal BCG infection [55]. Another study revealed that mouse IgA monoclonal antibody against the Mtb antigen Acr reduced early pulmonary Mtb infection in mice [56]. It was also reported that passive administration of purified secretory IgA from human colostrum reduced the pneumonic area in a murine infection model [24]. These findings indicate that IgA may play a pivotal role in the host's early defense against Mtb invasion in the respiratory tract.

Despite the great efforts that have been made to develop a novel vaccine that can effectively induce CMI to eliminate intracellular Mtb bacilli, these vaccine candidates have failed to induce better protection than BCG. As recent studies extensively demonstrated that mucosal immune responses are important in protecting the host from Mtb infection [57, 58], vaccine strategies that attempt to enhance mucosal immunity should be included in future TB vaccine development efforts. While the results of the present study suggest a possible role of specific antibodies in TB protection, and thus the benefit of potential inclusion of some of the investigated antigens in the development of a future vaccine candidate, the results are still not fully conclusive at this point, highlighting the need for further research in this area.

## 5. Conclusion

To our knowledge, this is the first human study to investigate the relationship between the kinetics of humoral antibody to various Mtb antigens and the clinical disease status during the treatment. The correlation between humoral immunity and bacterial and clinical parameters was analyzed for the first time. Antigen-specific IgA suppresses inflammatory responses in active tuberculosis patients. The data in this study support the inclusion of strategies that elicit humoral immunity when developing vaccines against tuberculosis.

## Figures and Tables

**Table 1:** Individual patient characteristics. Severity of smear at entry was subdivided as 0 (no acid-fast bacilli (AFB) on smear), ± (1-2 AFB per 300 fields), 1+ (1–9 AFB per 100 fields), 2+ (more than 10 AFB per 100 fields), and 3+ (more than 10 AFB per fields).

ID	Age	Sex	Smear at entry	CRP at entry (mg/dl)	CRP after 60 days (mg/dl)
1	83	M	1	3.68	0
2	63	F	3	2.39	0.39
3	61	F	2	10.4	3.55
4	64	M	2	0.17	0
5	36	F	2	4.93	1.14
6	43	M	3	0.6	0.18
7	66	M	3	12.79	13.09
8	33	F	2	0.02	0.02
9	56	M	2	0.04	0.05
10	63	M	3	10.45	8.7
11	71	M	3	0.69	0
12	71	M	3	11.31	3.91
13	68	M	3	5.12	5.27
14	69	F	2	2.4	0.17
15	57	M	3	7.36	2.99
16	54	M	3	16.52	1.15
17	83	M	2	2.71	1.28
18	32	M	2	0.11	0.11
19	47	M	3	11.47	4.67
20	46	M	2	0.44	0.06
21	64	M	2	0.85	1.59
22	51	M	2	1.78	0.2
23	68	M	3	1.9	0.83
24	78	F	3	4.2	1.73
25	76	F	2	2.87	1.76
26	38	F	3	0.56	0.5
27	27	F	1	4.73	0.27
28	34	F	2	1.43	0.01
29	54	M	2	6.12	2.8
30	62	F	3	18.63	6.52
31	59	M	2	6.12	7.92
32	27	M	3	4.04	1.66
33	31	M	1	1.76	0.05

## Supplementary Materials

Supplementary 1 Supplemental Figure 1: (A) IgG responses to Mtb antigens. Levels of serum IgG against 7 antigens (ESAT-6, CFP-10, MDP1, Ag85A, Acr, HBHA, and HrpA) before treatment (labeled as “before”) and after treatment (labeled as “after”) were analyzed by ELISA. Data shown are the average of triplicate experiments. Vertical lines: mean values. (B) IgG avidity index to Mtb antigens. Levels of serum IgG avidity index against 7 antigens (ESAT-6, CFP-10, MDP1, Ag85A, Acr, HBHA, and HrpA) before treatment (labeled as “before”) and after treatment (labeled as “after”) were analyzed by Urea ELISA. Data shown are the average of triplicate experiments. Vertical lines: mean values, ^∗∗^ *p* < 0.01 and ^∗^ *p* < 0.05. Supplemental Figure 2: (A) IgA responses to Mtb antigens. Levels of serum IgA against 7 antigens (ESAT-6, CFP-10, MDP1, Ag85A, Acr, HBHA, and HrpA) before treatment (labeled as “before”) and after treatment (labeled as “after”) were analyzed by ELISA. Data shown are the average of triplicate experiments. Vertical lines: mean values. (B) IgA avidity index to Mtb antigens. Levels of serum IgA avidity index against 7 antigens (ESAT-6, CFP-10, MDP1, Ag85A, Acr, HBHA, and HrpA) before treatment (labeled as “before”) and after treatment (labeled as “after”) were analyzed by Urea ELISA. Data shown are the average of triplicate experiments. Vertical lines: mean values.

Supplementary 2 Supplemental Table 1: antibody responses in individuals.
