# Supplementary material for: Longitudinal Evaluation of Humoral Immunity and Bacterial and Clinical Parameters Reveals That Antigen-Specific Antibodies Suppress Inflammatory Responses in Active Tuberculosis Patients
Source: J Immunol Res. 2018 Jul 4;2018:4928757. doi: 10.1155/2018/4928757 (PMC6057312; doi:10.1155/2018/4928757)
Supplement: Supplementary 2 — Supplemental Table 1: antibody responses in individuals. [file 4928757.f2.pdf]

Supplemental table 1. Antibody responses in individuals

ID	IgG level-before treatment						
	ESAT-6	CFP-10	MDP1	Ag85A	Acr	HBHA	HrpA
01	0.387	0.854	1.286	1.137	0.379	0.615	0.220
02	0.109	0.108	0.878	0.130	0.113	0.057	0.090
03	0.404	0.572	1.534	0.931	0.319	0.495	0.162
04	0.120	0.385	2.368	1.260	0.376	0.176	0.115
05	0.203	0.592	2.456	0.310	0.349	0.044	0.080
06	0.032	0.114	0.813	0.255	0.094	0.924	0.578
07	0.533	1.359	1.522	1.309	0.499	1.249	0.924
08	0.095	0.304	1.127	0.212	0.148	0.561	0.150
09	0.144	0.177	1.701	0.125	0.140	0.458	0.273
10	0.095	0.190	0.806	0.178	0.113	0.311	0.315
11	0.052	0.305	1.441	0.302	0.437	0.465	0.578
12	0.029	0.156	1.336	0.167	0.002	0.322	0.320
13	0.261	0.654	2.119	1.030	0.418	1.481	0.635
14	0.175	0.288	1.192	0.206	0.053	0.259	0.332
15	0.182	0.624	1.416	0.465	0.251	1.069	0.962
16	0.101	0.386	1.412	0.205	1.079	0.578	0.745
17	0.239	0.350	2.205	2.133	0.408	0.342	0.123
18	0.010	0.625	1.644	0.265	0.216	0.308	0.136
19	0.045	0.616	1.105	0.260	0.123	0.635	0.234
20	0.023	0.358	1.394	0.069	0.251	0.331	0.647
21	0.036	1.775	0.985	0.225	0.208	0.291	0.238
22	0.039	0.447	0.584	0.150	0.065	0.197	0.180
23	0.186	1.571	1.350	0.943	0.633	1.271	1.150
24	0.045	0.337	0.404	0.029	0.030	0.325	0.131
25	0.053	0.481	0.851	0.087	0.071	0.471	0.193
26	1.243	2.838	2.745	2.301	2.502	1.812	1.488
27	0.059	0.819	1.539	0.188	0.157	0.253	0.370
28	0.068	1.213	1.729	0.173	0.212	0.149	0.110
29	0.088	0.162	0.150	-0.004	-0.001	0.449	0.568
30	0.127	0.350	0.059	0.047	0.045	0.836	0.152
31	0.201	0.701	1.479	0.252	0.243	0.512	0.409
32	0.043	1.323	0.548	0.005	0.029	0.121	0.123
33	0.159	1.179	1.398	0.302	0.178	0.134	0.199

Supplemental table 1. continued

ID	IgG level-after treatment						
	ESAT-6	CFP-10	MDP1	Ag85A	Acr	HBHA	HrpA
01	0.240	0.559	1.401	0.751	0.260	0.312	0.170
02	0.086	0.094	0.630	0.127	0.131	0.044	0.080
03	0.754	0.536	2.152	1.288	0.636	0.638	0.085
04	0.099	0.335	2.109	1.344	0.376	0.222	0.126
5	0.116	0.137	2.283	0.258	0.288	0.495	0.162
6	0.066	0.131	0.955	0.184	0.129	0.552	0.464
7	0.410	0.923	1.583	0.890	0.362	0.875	0.716
8	0.039	0.243	0.931	0.127	0.093	0.546	0.134
9	0.144	0.126	1.434	0.202	0.132	0.335	0.217
10	-0.002	0.099	1.320	0.088	0.038	0.466	0.124
11	0.081	0.492	1.887	0.531	0.503	0.668	0.620
12	0.020	0.253	0.948	0.298	-0.009	0.207	0.461
13	0.234	0.681	2.009	0.971	0.594	1.860	0.783
14	0.132	0.235	0.841	0.199	0.016	0.107	0.229
15	0.170	0.338	1.050	0.296	0.201	0.892	0.706
16	0.105	0.257	1.749	0.199	0.121	0.714	0.773
17	0.166	0.313	2.147	1.828	0.273	0.112	0.110
18	0.024	0.684	1.710	0.246	0.206	0.350	0.148
19	0.058	0.569	0.914	0.215	0.073	0.793	0.168
20	0.015	0.419	1.360	0.078	0.210	0.090	0.437
21	0.017	0.840	0.691	0.120	0.020	0.176	0.129
22	0.031	0.571	0.559	0.126	0.084	0.196	0.204
23	0.178	1.369	1.193	0.595	0.381	0.940	0.713
24	0.169	0.326	0.537	0.030	0.009	0.750	0.148
25	0.069	0.415	0.614	0.121	0.077	0.465	0.190
26	0.904	2.783	2.336	1.469	1.251	1.711	1.216
27	0.075	0.876	1.735	0.206	0.127	0.331	0.423
28	0.022	1.460	1.867	0.158	0.166	0.133	0.021
29	0.108	0.293	0.363	0.012	0.018	0.445	0.441
30	0.116	0.419	0.627	0.211	0.064	0.814	0.204
31	0.146	0.666	1.469	0.303	0.162	0.442	0.336
32	0.070	1.419	1.733	0.044	0.083	0.173	0.102
33	0.029	1.083	1.347	0.341	0.244	0.140	0.240

Supplemental table 1. continued

ID	IgG Avidity level-before treatment						
	ESAT-6	CFP-10	MDP1	Ag85A	Acr	HBHA	HrpA
1	0.877	0.786	0.558	0.828	0.740	0.798	0.625
2	0.558	0.623	0.379	0.664	0.341	0.219	0.335
3	0.711	0.429	0.541	0.705	0.329	0.590	0.657
4	0.579	0.488	0.797	0.824	0.758	0.327	0.734
5	0.686	0.160	0.664	0.680	0.638	0.253	0.981
6	1.031	0.461	0.558	0.580	0.553	0.863	0.876
7	0.455	0.709	0.667	0.710	0.402	0.832	0.880
8	0.163	0.877	0.391	0.686	0.568	0.766	0.455
9	0.632	0.777	0.575	0.916	1.300	0.465	0.564
10	0.953	0.433	0.429	0.265	0.447	0.559	0.633
11	0.728	0.548	0.353	0.504	0.223	0.805	0.448
12	1.448	0.455	0.508	0.977	1.121	0.620	0.631
13	0.289	0.780	0.597	0.898	0.794	0.749	0.632
14	0.683	0.439	0.557	0.331	0.676	0.294	0.246
15	1.505	0.686	0.554	0.754	0.599	0.522	0.622
16	0.515	0.187	0.398	0.408	0.035	0.600	0.616
17	0.467	0.635	0.689	0.440	0.598	0.466	0.755
18	0.200	0.748	0.760	0.637	0.620	0.839	0.900
19	0.511	0.623	0.621	0.135	0.565	0.893	0.672
20	0.111	0.916	0.685	0.783	0.637	0.805	0.349
21	0.319	0.798	0.736	0.673	0.178	0.568	0.269
22	0.603	0.682	0.618	0.490	0.395	0.160	0.348
23	0.796	0.886	0.622	0.965	0.379	0.782	0.816
24	0.967	0.698	0.529	1.379	1.267	1.057	0.706
25	0.566	0.905	0.661	1.532	0.418	0.644	0.764
26	1.006	0.985	0.846	0.676	0.576	0.814	0.577
27	0.839	0.607	0.551	0.543	0.476	0.313	0.456
28	1.118	0.759	0.716	0.575	0.795	0.523	0.036
29	0.257	0.300	0.261	1.000	0.500	0.275	0.412
30	0.736	0.550	0.395	0.979	0.090	0.570	0.549
31	0.490	0.534	0.447	0.336	0.320	0.570	0.479
32	0.329	0.681	0.173	0.778	0.017	0.419	0.363
33	0.038	0.549	0.472	0.599	0.256	0.461	0.242

Supplemental table 1. continued

ID	IgG Avidity level-after treatment						
	ESAT-6	CFP-10	MDP1	Ag85A	Acr	HBHA	HrpA
1	0.765	0.549	0.487	0.623	0.526	0.750	0.588
2	0.760	0.862	0.672	1.071	0.483	0.253	0.981
3	0.086	0.151	0.197	0.106	0.099	0.780	1.473
4	0.727	0.553	0.686	0.672	0.574	0.140	0.510
5	0.671	0.458	0.615	0.601	0.581	0.590	0.657
6	0.818	0.529	0.504	0.564	0.389	1.072	0.763
7	0.184	0.698	0.499	0.728	0.463	0.755	0.780
8	1.494	0.414	0.386	1.228	0.925	0.755	0.183
9	1.355	0.745	0.477	0.635	0.715	0.348	0.707
10	1.239	0.335	0.103	0.044	0.312	0.346	0.474
11	1.198	0.575	0.364	0.354	0.304	0.651	0.514
12	1.305	0.311	0.483	0.225	0.952	0.454	0.651
13	0.923	0.895	0.577	1.012	0.478	0.744	0.594
14	0.231	0.318	0.409	0.174	0.813	0.065	0.077
15	0.354	0.646	0.481	0.550	0.381	0.664	0.565
16	0.684	0.530	0.495	0.392	0.355	0.762	0.634
17	0.964	0.543	0.682	0.344	0.729	0.571	0.573
18	0.063	0.576	0.613	0.460	0.791	0.774	0.861
19	1.009	0.526	0.574	0.053	0.159	0.748	0.601
20	0.733	0.606	0.598	0.378	0.462	0.039	0.233
21	0.061	0.742	0.737	0.979	1.000	0.595	0.339
22	1.115	0.384	0.481	0.912	0.048	0.181	0.402
23	0.994	0.868	0.597	1.030	0.617	0.813	0.809
24	0.453	0.848	0.551	1.288	1.000	0.885	0.733
25	0.688	0.639	0.494	0.740	0.558	0.626	0.503
26	0.757	0.863	0.735	0.612	0.620	0.823	0.629
27	0.967	0.665	0.669	0.586	0.646	0.530	0.561
28	0.591	0.725	0.690	0.308	0.622	0.380	0.143
29	0.222	0.311	0.397	0.261	0.111	0.178	0.490
30	1.315	0.578	0.344	0.727	1.273	0.801	0.505
31	0.409	0.550	0.418	0.195	0.279	0.891	0.323
32	0.331	0.780	0.133	0.632	0.109	0.350	0.456
33	0.862	0.585	0.434	0.581	0.348	0.450	0.315

Supplemental table 1. continued

ID	IgA ELISA Index-before treatment						
	ESAT-6	CFP-10	MDP1	Ag85A	Acr	HBHA	HrpA
1	1.217	2.981	2.225	5.399	1.768	1.173	1.458
2	0.983	1.158	0.861	1.004	0.838	0.952	1.092
3	0.336	0.478	0.545	0.423	0.356	0.909	0.827
4	1.136	1.222	1.230	0.733	0.509	0.725	0.993
5	0.654	2.072	2.794	0.801	0.476	0.855	0.887
6	1.322	1.198	1.908	0.858	0.733	1.179	1.177
7	1.764	2.578	8.830	2.022	1.696	0.535	0.654
8	2.540	3.657	3.767	2.977	1.948	1.104	0.729
9	1.196	1.376	2.468	1.279	1.086	1.885	1.243
10	0.471	1.195	0.869	0.784	0.359	1.252	0.947
11	1.176	2.132	2.004	1.492	0.866	0.876	0.949
12	0.582	2.104	3.089	1.166	0.894	0.854	0.698
13	1.723	4.614	7.193	2.564	1.265	3.656	2.293
14	0.848	2.546	4.432	1.930	0.932	1.160	1.259
15	2.249	3.081	4.314	2.516	1.982	0.814	0.800
16	0.768	0.957	1.004	0.905	0.741	0.901	0.836
17	1.491	1.595	1.109	1.781	0.847	3.364	1.993
18	0.833	0.823	1.486	1.095	0.532	0.939	0.809
19	1.069	1.774	2.991	0.633	0.608	0.816	1.056
20	0.711	0.973	1.011	0.790	0.702	2.575	1.827
21	1.668	2.868	3.350	1.205	0.866	2.227	1.374
22	1.982	3.223	2.993	2.936	1.620	1.246	1.113
23	2.154	4.693	5.090	2.126	1.494	1.551	1.609
24	0.478	1.473	0.919	0.662	0.625	1.740	1.026
25	1.470	1.760	1.655	1.150	1.165	1.682	1.566
26	0.651	3.965	1.262	0.857	0.753	2.443	1.974
27	1.816	2.321	1.877	0.730	0.781	0.624	0.768
28	0.879	1.475	1.214	1.028	0.759	0.901	0.990
29	1.156	1.883	1.650	0.730	0.913	2.772	1.352
30	1.012	1.924	1.184	1.295	1.149	0.999	0.722
31	1.165	2.698	2.239	0.953	1.224	0.798	0.880
32	0.699	1.446	0.859	0.843	0.653	1.360	0.700
33	0.732	2.171	1.413	1.082	0.768	2.412	1.188

Supplemental table 1. continued

ID	IgA ELISA Index-after treatment						
	ESAT-6	CFP-10	MDP1	Ag85A	Acr	HBHA	HrpA
1	1.528	3.207	3.314	5.771	1.570	1.234	1.586
2	0.419	0.484	0.589	0.612	0.582	1.306	1.221
3	1.410	1.287	1.586	1.401	0.671	1.193	1.158
4	0.418	0.668	0.878	0.507	0.410	0.750	0.860
5	1.278	3.161	4.046	1.260	0.836	1.886	1.143
6	0.398	0.873	1.542	0.556	0.473	1.614	1.196
7	0.395	0.544	2.681	0.369	0.325	0.396	0.542
8	2.142	2.894	3.155	2.315	1.568	3.342	1.594
9	0.997	1.427	2.640	1.852	1.136	1.115	0.868
10	1.727	2.008	1.856	1.366	0.748	1.276	0.982
11	1.106	5.419	1.435	2.240	0.602	1.041	0.626
12	1.689	5.059	5.823	2.365	2.056	1.454	1.171
13	0.960	1.654	2.928	1.321	0.757	2.576	1.618
14	1.330	2.543	4.348	2.042	1.313	0.835	0.944
15	1.241	3.119	4.691	2.015	1.602	1.090	0.825
16	0.594	0.737	1.202	0.593	0.521	2.396	0.867
17	1.081	1.752	1.385	2.021	0.960	2.332	1.398
18	0.834	0.699	1.378	0.447	0.504	0.859	0.683
19	0.701	1.766	2.957	0.666	0.619	1.248	1.389
20	1.619	1.807	2.415	1.479	0.970	3.696	1.857
21	1.374	1.502	2.053	1.000	0.640	1.522	0.927
22	1.679	2.227	2.159	2.091	1.398	1.146	1.013
23	1.223	3.874	4.079	1.956	1.300	1.747	1.652
24	0.727	2.348	1.983	1.130	1.265	2.050	1.285
25	1.163	2.265	2.045	1.063	1.220	1.319	1.352
26	1.722	7.864	2.145	1.604	1.157	1.941	1.544
27	0.526	1.383	1.359	0.515	0.559	0.754	0.744
28	1.231	2.497	1.947	0.962	0.979	2.179	1.326
29	1.260	2.130	1.520	0.728	0.883	2.026	1.299
30	1.012	1.748	1.303	0.828	0.699	0.598	0.570
31	0.819	2.184	2.123	1.003	1.024	0.711	0.754
32	0.730	1.441	1.192	0.925	0.689	3.006	1.378
33	0.563	1.181	0.951	0.857	0.641	1.003	0.673

Supplemental table 1. continued

ID	IgA Avidity level-before treatment						
	ESAT-6	CFP-10	MDP1	Ag85A	Acr	HBHA	HrpA
1	0.858	1.119	0.913	1.236	1.203	1.022	0.888
2	0.784	0.792	0.702	0.539	0.647	0.841	0.976
3	1.316	0.924	0.944	1.063	1.113	0.738	0.775
4	0.715	0.484	0.307	0.459	0.814	0.678	0.707
5	0.829	0.549	0.394	0.580	0.767	1.523	1.093
6	0.848	0.697	0.598	0.609	0.663	1.169	1.156
7	0.730	0.588	0.204	0.624	0.639	0.633	0.940
8	0.569	0.672	0.531	0.713	0.689	1.213	1.487
9	0.833	0.742	0.735	0.803	0.826	0.864	1.039
10	0.814	0.598	0.906	0.746	1.254	0.519	0.809
11	0.766	0.577	0.540	0.543	0.774	0.636	0.677
12	1.113	0.404	0.420	0.679	0.652	1.008	1.188
13	1.049	0.409	0.395	0.629	0.846	0.362	0.757
14	0.668	0.288	0.291	0.336	0.413	0.639	0.733
15	0.821	0.668	0.483	0.712	0.553	0.882	1.187
16	1.316	0.948	1.167	1.081	1.193	0.882	0.882
17	0.631	0.695	0.814	0.898	1.019	1.692	0.765
18	1.069	0.733	0.585	0.425	0.825	0.357	0.460
19	0.988	0.735	0.547	0.813	0.753	0.983	0.779
20	1.100	0.725	0.756	0.926	0.491	0.542	0.872
21	0.785	0.388	0.459	0.515	0.582	0.680	0.710
22	0.835	0.566	0.535	0.855	0.640	0.689	0.584
23	0.834	0.604	0.578	1.017	0.818	1.033	0.850
24	0.575	0.361	0.441	0.426	0.345	1.034	0.696
25	1.374	1.126	1.119	0.587	0.606	1.557	0.680
26	0.624	0.554	0.475	0.725	0.574	0.539	0.566
27	0.814	0.676	1.006	1.037	0.950	0.503	0.395
28	1.030	0.921	1.240	0.907	1.278	0.733	0.568
29	0.720	0.697	0.631	1.568	0.741	0.776	0.790
30	1.143	0.895	1.106	0.820	0.578	1.213	0.726
31	0.907	0.402	0.442	0.505	0.472	0.705	0.540
32	1.095	0.679	0.865	1.144	1.091	0.941	0.956
33	1.136	0.585	0.610	0.667	0.666	0.794	0.874

Supplemental table 1. Antibody responses in individuals

ID	IgG level-before treatment						
	ESAT-6	CFP-10	MDP1	Ag85A	Acr	HBHA	HrpA
01	0.387	0.854	1.286	1.137	0.379	0.615	0.220
02	0.109	0.108	0.878	0.130	0.113	0.057	0.090
03	0.404	0.572	1.534	0.931	0.319	0.495	0.162
04	0.120	0.385	2.368	1.260	0.376	0.176	0.115
05	0.203	0.592	2.456	0.310	0.349	0.044	0.080
06	0.032	0.114	0.813	0.255	0.094	0.924	0.578
07	0.533	1.359	1.522	1.309	0.499	1.249	0.924
08	0.095	0.304	1.127	0.212	0.148	0.561	0.150
09	0.144	0.177	1.701	0.125	0.140	0.458	0.273
10	0.095	0.190	0.806	0.178	0.113	0.311	0.315
11	0.052	0.305	1.441	0.302	0.437	0.465	0.578
12	0.029	0.156	1.336	0.167	0.002	0.322	0.320
13	0.261	0.654	2.119	1.030	0.418	1.481	0.635
14	0.175	0.288	1.192	0.206	0.053	0.259	0.332
15	0.182	0.624	1.416	0.465	0.251	1.069	0.962
16	0.101	0.386	1.412	0.205	1.079	0.578	0.745
17	0.239	0.350	2.205	2.133	0.408	0.342	0.123
18	0.010	0.625	1.644	0.265	0.216	0.308	0.136
19	0.045	0.616	1.105	0.260	0.123	0.635	0.234
20	0.023	0.358	1.394	0.069	0.251	0.331	0.647
21	0.036	1.775	0.985	0.225	0.208	0.291	0.238
22	0.039	0.447	0.584	0.150	0.065	0.197	0.180
23	0.186	1.571	1.350	0.943	0.633	1.271	1.150
24	0.045	0.337	0.404	0.029	0.030	0.325	0.131
25	0.053	0.481	0.851	0.087	0.071	0.471	0.193
26	1.243	2.838	2.745	2.301	2.502	1.812	1.488
27	0.059	0.819	1.539	0.188	0.157	0.253	0.370
28	0.068	1.213	1.729	0.173	0.212	0.149	0.110
29	0.088	0.162	0.150	-0.004	-0.001	0.449	0.568
30	0.127	0.350	0.059	0.047	0.045	0.836	0.152
31	0.201	0.701	1.479	0.252	0.243	0.512	0.409
32	0.043	1.323	0.548	0.005	0.029	0.121	0.123
33	0.159	1.179	1.398	0.302	0.178	0.134	0.199

Supplemental table 1. continued

ID	IgG level-after treatment						
	ESAT-6	CFP-10	MDP1	Ag85A	Acr	HBHA	HrpA
01	0.240	0.559	1.401	0.751	0.260	0.312	0.170
02	0.086	0.094	0.630	0.127	0.131	0.044	0.080
03	0.754	0.536	2.152	1.288	0.636	0.638	0.085
04	0.099	0.335	2.109	1.344	0.376	0.222	0.126
5	0.116	0.137	2.283	0.258	0.288	0.495	0.162
6	0.066	0.131	0.955	0.184	0.129	0.552	0.464
7	0.410	0.923	1.583	0.890	0.362	0.875	0.716
8	0.039	0.243	0.931	0.127	0.093	0.546	0.134
9	0.144	0.126	1.434	0.202	0.132	0.335	0.217
10	-0.002	0.099	1.320	0.088	0.038	0.466	0.124
11	0.081	0.492	1.887	0.531	0.503	0.668	0.620
12	0.020	0.253	0.948	0.298	-0.009	0.207	0.461
13	0.234	0.681	2.009	0.971	0.594	1.860	0.783
14	0.132	0.235	0.841	0.199	0.016	0.107	0.229
15	0.170	0.338	1.050	0.296	0.201	0.892	0.706
16	0.105	0.257	1.749	0.199	0.121	0.714	0.773
17	0.166	0.313	2.147	1.828	0.273	0.112	0.110
18	0.024	0.684	1.710	0.246	0.206	0.350	0.148
19	0.058	0.569	0.914	0.215	0.073	0.793	0.168
20	0.015	0.419	1.360	0.078	0.210	0.090	0.437
21	0.017	0.840	0.691	0.120	0.020	0.176	0.129
22	0.031	0.571	0.559	0.126	0.084	0.196	0.204
23	0.178	1.369	1.193	0.595	0.381	0.940	0.713
24	0.169	0.326	0.537	0.030	0.009	0.750	0.148
25	0.069	0.415	0.614	0.121	0.077	0.465	0.190
26	0.904	2.783	2.336	1.469	1.251	1.711	1.216
27	0.075	0.876	1.735	0.206	0.127	0.331	0.423
28	0.022	1.460	1.867	0.158	0.166	0.133	0.021
29	0.108	0.293	0.363	0.012	0.018	0.445	0.441
30	0.116	0.419	0.627	0.211	0.064	0.814	0.204
31	0.146	0.666	1.469	0.303	0.162	0.442	0.336
32	0.070	1.419	1.733	0.044	0.083	0.173	0.102
33	0.029	1.083	1.347	0.341	0.244	0.140	0.240

Supplemental table 1. continued

ID	IgG Avidity level-before treatment						
	ESAT-6	CFP-10	MDP1	Ag85A	Acr	HBHA	HrpA
1	0.877	0.786	0.558	0.828	0.740	0.798	0.625
2	0.558	0.623	0.379	0.664	0.341	0.219	0.335
3	0.711	0.429	0.541	0.705	0.329	0.590	0.657
4	0.579	0.488	0.797	0.824	0.758	0.327	0.734
5	0.686	0.160	0.664	0.680	0.638	0.253	0.981
6	1.031	0.461	0.558	0.580	0.553	0.863	0.876
7	0.455	0.709	0.667	0.710	0.402	0.832	0.880
8	0.163	0.877	0.391	0.686	0.568	0.766	0.455
9	0.632	0.777	0.575	0.916	1.300	0.465	0.564
10	0.953	0.433	0.429	0.265	0.447	0.559	0.633
11	0.728	0.548	0.353	0.504	0.223	0.805	0.448
12	1.448	0.455	0.508	0.977	1.121	0.620	0.631
13	0.289	0.780	0.597	0.898	0.794	0.749	0.632
14	0.683	0.439	0.557	0.331	0.676	0.294	0.246
15	1.505	0.686	0.554	0.754	0.599	0.522	0.622
16	0.515	0.187	0.398	0.408	0.035	0.600	0.616
17	0.467	0.635	0.689	0.440	0.598	0.466	0.755
18	0.200	0.748	0.760	0.637	0.620	0.839	0.900
19	0.511	0.623	0.621	0.135	0.565	0.893	0.672
20	0.111	0.916	0.685	0.783	0.637	0.805	0.349
21	0.319	0.798	0.736	0.673	0.178	0.568	0.269
22	0.603	0.682	0.618	0.490	0.395	0.160	0.348
23	0.796	0.886	0.622	0.965	0.379	0.782	0.816
24	0.967	0.698	0.529	1.379	1.267	1.057	0.706
25	0.566	0.905	0.661	1.532	0.418	0.644	0.764
26	1.006	0.985	0.846	0.676	0.576	0.814	0.577
27	0.839	0.607	0.551	0.543	0.476	0.313	0.456
28	1.118	0.759	0.716	0.575	0.795	0.523	0.036
29	0.257	0.300	0.261	1.000	0.500	0.275	0.412
30	0.736	0.550	0.395	0.979	0.090	0.570	0.549
31	0.490	0.534	0.447	0.336	0.320	0.570	0.479
32	0.329	0.681	0.173	0.778	0.017	0.419	0.363
33	0.038	0.549	0.472	0.599	0.256	0.461	0.242

Supplemental table 1. continued

ID	IgG Avidity level-after treatment						
	ESAT-6	CFP-10	MDP1	Ag85A	Acr	HBHA	HrpA
1	0.765	0.549	0.487	0.623	0.526	0.750	0.588
2	0.760	0.862	0.672	1.071	0.483	0.253	0.981
3	0.086	0.151	0.197	0.106	0.099	0.780	1.473
4	0.727	0.553	0.686	0.672	0.574	0.140	0.510
5	0.671	0.458	0.615	0.601	0.581	0.590	0.657
6	0.818	0.529	0.504	0.564	0.389	1.072	0.763
7	0.184	0.698	0.499	0.728	0.463	0.755	0.780
8	1.494	0.414	0.386	1.228	0.925	0.755	0.183
9	1.355	0.745	0.477	0.635	0.715	0.348	0.707
10	1.239	0.335	0.103	0.044	0.312	0.346	0.474
11	1.198	0.575	0.364	0.354	0.304	0.651	0.514
12	1.305	0.311	0.483	0.225	0.952	0.454	0.651
13	0.923	0.895	0.577	1.012	0.478	0.744	0.594
14	0.231	0.318	0.409	0.174	0.813	0.065	0.077
15	0.354	0.646	0.481	0.550	0.381	0.664	0.565
16	0.684	0.530	0.495	0.392	0.355	0.762	0.634
17	0.964	0.543	0.682	0.344	0.729	0.571	0.573
18	0.063	0.576	0.613	0.460	0.791	0.774	0.861
19	1.009	0.526	0.574	0.053	0.159	0.748	0.601
20	0.733	0.606	0.598	0.378	0.462	0.039	0.233
21	0.061	0.742	0.737	0.979	1.000	0.595	0.339
22	1.115	0.384	0.481	0.912	0.048	0.181	0.402
23	0.994	0.868	0.597	1.030	0.617	0.813	0.809
24	0.453	0.848	0.551	1.288	1.000	0.885	0.733
25	0.688	0.639	0.494	0.740	0.558	0.626	0.503
26	0.757	0.863	0.735	0.612	0.620	0.823	0.629
27	0.967	0.665	0.669	0.586	0.646	0.530	0.561
28	0.591	0.725	0.690	0.308	0.622	0.380	0.143
29	0.222	0.311	0.397	0.261	0.111	0.178	0.490
30	1.315	0.578	0.344	0.727	1.273	0.801	0.505
31	0.409	0.550	0.418	0.195	0.279	0.891	0.323
32	0.331	0.780	0.133	0.632	0.109	0.350	0.456
33	0.862	0.585	0.434	0.581	0.348	0.450	0.315

Supplemental table 1. continued

ID	IgA ELISA Index-before treatment						
	ESAT-6	CFP-10	MDP1	Ag85A	Acr	HBHA	HrpA
1	1.217	2.981	2.225	5.399	1.768	1.173	1.458
2	0.983	1.158	0.861	1.004	0.838	0.952	1.092
3	0.336	0.478	0.545	0.423	0.356	0.909	0.827
4	1.136	1.222	1.230	0.733	0.509	0.725	0.993
5	0.654	2.072	2.794	0.801	0.476	0.855	0.887
6	1.322	1.198	1.908	0.858	0.733	1.179	1.177
7	1.764	2.578	8.830	2.022	1.696	0.535	0.654
8	2.540	3.657	3.767	2.977	1.948	1.104	0.729
9	1.196	1.376	2.468	1.279	1.086	1.885	1.243
10	0.471	1.195	0.869	0.784	0.359	1.252	0.947
11	1.176	2.132	2.004	1.492	0.866	0.876	0.949
12	0.582	2.104	3.089	1.166	0.894	0.854	0.698
13	1.723	4.614	7.193	2.564	1.265	3.656	2.293
14	0.848	2.546	4.432	1.930	0.932	1.160	1.259
15	2.249	3.081	4.314	2.516	1.982	0.814	0.800
16	0.768	0.957	1.004	0.905	0.741	0.901	0.836
17	1.491	1.595	1.109	1.781	0.847	3.364	1.993
18	0.833	0.823	1.486	1.095	0.532	0.939	0.809
19	1.069	1.774	2.991	0.633	0.608	0.816	1.056
20	0.711	0.973	1.011	0.790	0.702	2.575	1.827
21	1.668	2.868	3.350	1.205	0.866	2.227	1.374
22	1.982	3.223	2.993	2.936	1.620	1.246	1.113
23	2.154	4.693	5.090	2.126	1.494	1.551	1.609
24	0.478	1.473	0.919	0.662	0.625	1.740	1.026
25	1.470	1.760	1.655	1.150	1.165	1.682	1.566
26	0.651	3.965	1.262	0.857	0.753	2.443	1.974
27	1.816	2.321	1.877	0.730	0.781	0.624	0.768
28	0.879	1.475	1.214	1.028	0.759	0.901	0.990
29	1.156	1.883	1.650	0.730	0.913	2.772	1.352
30	1.012	1.924	1.184	1.295	1.149	0.999	0.722
31	1.165	2.698	2.239	0.953	1.224	0.798	0.880
32	0.699	1.446	0.859	0.843	0.653	1.360	0.700
33	0.732	2.171	1.413	1.082	0.768	2.412	1.188

Supplemental table 1. continued

ID	IgA ELISA Index-after treatment						
	ESAT-6	CFP-10	MDP1	Ag85A	Acr	HBHA	HrpA
1	1.528	3.207	3.314	5.771	1.570	1.234	1.586
2	0.419	0.484	0.589	0.612	0.582	1.306	1.221
3	1.410	1.287	1.586	1.401	0.671	1.193	1.158
4	0.418	0.668	0.878	0.507	0.410	0.750	0.860
5	1.278	3.161	4.046	1.260	0.836	1.886	1.143
6	0.398	0.873	1.542	0.556	0.473	1.614	1.196
7	0.395	0.544	2.681	0.369	0.325	0.396	0.542
8	2.142	2.894	3.155	2.315	1.568	3.342	1.594
9	0.997	1.427	2.640	1.852	1.136	1.115	0.868
10	1.727	2.008	1.856	1.366	0.748	1.276	0.982
11	1.106	5.419	1.435	2.240	0.602	1.041	0.626
12	1.689	5.059	5.823	2.365	2.056	1.454	1.171
13	0.960	1.654	2.928	1.321	0.757	2.576	1.618
14	1.330	2.543	4.348	2.042	1.313	0.835	0.944
15	1.241	3.119	4.691	2.015	1.602	1.090	0.825
16	0.594	0.737	1.202	0.593	0.521	2.396	0.867
17	1.081	1.752	1.385	2.021	0.960	2.332	1.398
18	0.834	0.699	1.378	0.447	0.504	0.859	0.683
19	0.701	1.766	2.957	0.666	0.619	1.248	1.389
20	1.619	1.807	2.415	1.479	0.970	3.696	1.857
21	1.374	1.502	2.053	1.000	0.640	1.522	0.927
22	1.679	2.227	2.159	2.091	1.398	1.146	1.013
23	1.223	3.874	4.079	1.956	1.300	1.747	1.652
24	0.727	2.348	1.983	1.130	1.265	2.050	1.285
25	1.163	2.265	2.045	1.063	1.220	1.319	1.352
26	1.722	7.864	2.145	1.604	1.157	1.941	1.544
27	0.526	1.383	1.359	0.515	0.559	0.754	0.744
28	1.231	2.497	1.947	0.962	0.979	2.179	1.326
29	1.260	2.130	1.520	0.728	0.883	2.026	1.299
30	1.012	1.748	1.303	0.828	0.699	0.598	0.570
31	0.819	2.184	2.123	1.003	1.024	0.711	0.754
32	0.730	1.441	1.192	0.925	0.689	3.006	1.378
33	0.563	1.181	0.951	0.857	0.641	1.003	0.673

Supplemental table 1. continued

ID	IgA Avidity level-before treatment						
	ESAT-6	CFP-10	MDP1	Ag85A	Acr	HBHA	HrpA
1	0.858	1.119	0.913	1.236	1.203	1.022	0.888
2	0.784	0.792	0.702	0.539	0.647	0.841	0.976
3	1.316	0.924	0.944	1.063	1.113	0.738	0.775
4	0.715	0.484	0.307	0.459	0.814	0.678	0.707
5	0.829	0.549	0.394	0.580	0.767	1.523	1.093
6	0.848	0.697	0.598	0.609	0.663	1.169	1.156
7	0.730	0.588	0.204	0.624	0.639	0.633	0.940
8	0.569	0.672	0.531	0.713	0.689	1.213	1.487
9	0.833	0.742	0.735	0.803	0.826	0.864	1.039
10	0.814	0.598	0.906	0.746	1.254	0.519	0.809
11	0.766	0.577	0.540	0.543	0.774	0.636	0.677
12	1.113	0.404	0.420	0.679	0.652	1.008	1.188
13	1.049	0.409	0.395	0.629	0.846	0.362	0.757
14	0.668	0.288	0.291	0.336	0.413	0.639	0.733
15	0.821	0.668	0.483	0.712	0.553	0.882	1.187
16	1.316	0.948	1.167	1.081	1.193	0.882	0.882
17	0.631	0.695	0.814	0.898	1.019	1.692	0.765
18	1.069	0.733	0.585	0.425	0.825	0.357	0.460
19	0.988	0.735	0.547	0.813	0.753	0.983	0.779
20	1.100	0.725	0.756	0.926	0.491	0.542	0.872
21	0.785	0.388	0.459	0.515	0.582	0.680	0.710
22	0.835	0.566	0.535	0.855	0.640	0.689	0.584
23	0.834	0.604	0.578	1.017	0.818	1.033	0.850
24	0.575	0.361	0.441	0.426	0.345	1.034	0.696
25	1.374	1.126	1.119	0.587	0.606	1.557	0.680
26	0.624	0.554	0.475	0.725	0.574	0.539	0.566
27	0.814	0.676	1.006	1.037	0.950	0.503	0.395
28	1.030	0.921	1.240	0.907	1.278	0.733	0.568
29	0.720	0.697	0.631	1.568	0.741	0.776	0.790
30	1.143	0.895	1.106	0.820	0.578	1.213	0.726
31	0.907	0.402	0.442	0.505	0.472	0.705	0.540
32	1.095	0.679	0.865	1.144	1.091	0.941	0.956
33	1.136	0.585	0.610	0.667	0.666	0.794	0.874

Supplemental table 1. continued

ID	IgA Avidity level-after treatment						
	ESAT-6	CFP-10	MDP1	Ag85A	Acr	HBHA	HrpA
1	0.781	1.249	0.866	1.252	1.303	0.824	0.759
2	0.829	1.317	0.811	0.674	0.818	0.845	0.809
3	0.502	0.612	0.419	0.519	0.727	0.585	0.688
4	1.132	0.609	0.365	0.710	0.773	0.794	0.722
5	0.864	0.695	0.438	0.729	0.820	1.086	0.765
6	1.298	1.017	0.963	1.176	1.278	1.206	0.800
7	0.639	0.967	0.264	0.875	0.905	0.864	0.752
8	0.591	0.568	0.491	1.138	0.645	0.322	0.599
9	0.993	0.719	0.566	0.571	0.798	1.027	1.225
10	0.476	0.451	0.517	0.531	0.660	0.645	0.975
11	0.695	0.394	0.922	0.579	1.683	0.493	0.783
12	1.133	0.308	0.405	0.654	0.600	0.737	0.868
13	0.596	0.383	0.317	0.433	0.505	0.234	0.590
14	1.067	0.763	0.676	0.832	0.887	1.079	1.191
15	1.158	0.735	0.512	0.807	0.707	0.759	0.884
16	0.881	1.098	0.598	1.077	1.223	0.690	0.941
17	1.007	0.787	0.804	0.971	1.156	0.994	0.543
18	1.279	0.855	0.521	0.935	0.906	0.639	0.695
19	1.402	0.767	0.596	1.037	0.952	0.492	0.501
20	1.159	0.874	0.775	0.994	0.882	0.515	0.938
21	0.456	0.479	0.430	0.574	0.717	0.953	1.120
22	1.131	0.755	0.758	0.920	0.831	1.382	1.308
23	1.221	0.638	0.615	1.058	0.876	0.779	0.624
24	1.575	0.588	0.593	0.455	0.404	1.359	0.832
25	0.771	0.757	0.727	0.595	0.470	1.187	0.611
26	0.989	1.225	0.832	1.214	1.014	0.787	0.739
27	1.352	0.853	0.883	0.909	0.782	0.385	0.283
28	1.015	0.563	0.719	0.895	0.862	0.648	0.544
29	0.577	0.458	0.811	0.863	0.771	0.830	0.693
30	1.134	0.963	0.923	1.301	1.038	1.234	1.051
31	0.234	0.165	0.240	0.299	0.271	1.013	0.930
32	1.094	0.819	0.635	0.807	0.888	0.576	0.659
33	1.460	0.917	1.022	1.080	1.271	1.210	1.124
